# Bridging the gap in mental health literacy: co-adapting and feasibility testing a digital intervention to improve mental health literacy amongst young people aged 12–14 in the UK

**DOI:** 10.3389/fpsyt.2025.1546109

**Published:** 2025-07-21

**Authors:** Emily Vicary, Helen Brierley, Jack Wilkinson, Melody Adesina, Penny Bee, Karina Lovell, Naz Uzun, Emily Bee, Helen Brooks

**Affiliations:** ^1^ Division of Nursing, Midwifery and Social Work, School of Health Sciences, Manchester Academic Health Centre, University of Manchester, Manchester, United Kingdom; ^2^ Centre for Biostatistics, Division of Population Health, Health Services Research and Primary Care, Manchester Academic Health Science Centre, University of Manchester, Manchester, United Kingdom; ^3^ Independent researcher, Manchester, United Kingdom

**Keywords:** anxiety, depression, digital application, mental health literacy, self-management, young people

## Abstract

**Background:**

Digital interventions have shown promise in enhancing mental health literacy among young people (YP). Initially developed in Indonesia, the Improving Mental Health Literacy Among Young People in Indonesia (IMPeTUs) intervention is a co-produced digital application designed to improve mental health literacy and self-management of anxiety and depression in YP. This study aimed to co-adapt the IMPeTUs intervention with YP in the UK and evaluate the feasibility of conducting a future definitive trial in education and community settings.

**Methods:**

The study consisted of two phases. Phase 1 involved co-adapting the intervention through consultations with 49 stakeholders, including YP, parents, and professionals, using principles of experience-based co-design. ‘Co-adapting’ and ‘co-creating’ refer to collaborative modification and development of intervention content with stakeholders. Then, Phase 2 was a multi-site, cluster-randomized feasibility trial with a nested mixed-methods process evaluation conducted at two community sites (N = 19, 12 completing 4-week post-intervention data and 11 completing 3-month follow-up). The ‘nested process evaluation’ assessed acceptability and engagement alongside the trial.

**Results:**

In Phase 1, the digital intervention was co-adapted with stakeholders to include expanded customization options, UK-specific content, and a new chapter on resilience, healthy relationships and self-care to improve engagement and support mental health literacy. Phase 2 showed the application was well-received, with YP appreciating its relatable storylines and problem-solving focus. Recruitment challenges and lengthy questionnaires highlighted the need for improved partnerships with schools and streamlined data collection. Despite these issues, the usability of the core design was validated, and recommendations to enhance engagement, such as reducing text and adding interactive features, were identified. Exploratory analyses suggested potential improvements in mental health literacy and well-being, although these results require confirmation in a well-powered trial.

**Conclusion:**

This study highlights the feasibility of co-adapting and implementing digital interventions to improve mental health literacy among YP within community settings. Future work should focus on refining recruitment strategies for school environments, streamlining data collection, and enhancing engagement features to ensure scalability and effectiveness in large-scale evaluations. These findings lay the foundation for further development and rigorous digital mental health literacy intervention assessment.

**Clinical trial registration:**

https://doi.org/10.1186/ISRCTN16116467, identifier ISRCTN16116467.

## 1 Introduction

Adolescence marks a period of physical, emotional and behavioural development significantly impacted by social factors (1). The combination of these complex developmental changes with encounters of adversity, peer conformity issues, media influence and identity exploration increases susceptibility to mental health issues. Approximately 15% of young people (YP) are diagnosed with a mental health disorder, constituting 13% of the overall burden of disease among individuals aged 10 to 19 years (2). In the UK alone, mental ill health is the most significant cause of disability, contributing up to 22.8% of the total burden, a greater burden than other major health diseases (3). Pre-pandemic analyses revealed that the estimated economic and societal cost of mental illness in England was £105.2 billion per year (4); however, emerging data suggest a further post-pandemic increase in burden and treatment need, specifically among YP (5).

The prevalence of diagnosable mental health conditions among children and YP has significantly increased in recent years. Over six years, NHS England reported an 8% rise in prevalence among children aged 8 to 16 and a 13% rise among YP aged 17 to 19, underscoring the urgent need for early intervention to address YP’s mental health (6). Early support is critical, as mental health problems that emerge during childhood and adolescence are more challenging and costly to treat and have a higher likelihood of becoming chronic if left unaddressed. Timely intervention not only mitigates long-term difficulties but also promotes healthier developmental outcomes. This increasing prevalence, compounded by the widespread and lasting impacts of the COVID-19 pandemic, highlights the pressing need for comprehensive and targeted approaches to support young people’s mental health.

The COVID-19 pandemic has exacerbated these challenges, with prevalence rates of mental health conditions surging in 2020 (7). YP experienced significant short-term effects, including heightened anxiety, loneliness, and disrupted routines. Over time, these challenges have contributed to long-term impacts such as developmental delays and an increased prevalence of chronic conditions like depression. This pandemic-related escalation has intensified the need for targeted mental health interventions. The long-term mental health impacts of the COVID-19 pandemic further emphasize the importance of preventative measures, which offer proactive and cost-effective solutions to address the growing needs of young people and reduce the risk of chronic conditions.

Preventative measures play a vital role in addressing YP’s growing mental health needs. These cost-effective, proactive approaches help mitigate mental health risks by reducing the likelihood of chronic conditions and promoting positive developmental outcomes (8). By bridging the gap between YP’s mental health needs and the limitations of existing interventions, preventative strategies provide an opportunity to build resilience and foster healthier future generations.

One promising avenue for such preventative strategies is improving mental health literacy. Mental health literacy can be defined as knowledge and beliefs about mental health conditions which help people prevent, recognize and manage problems (9). Research consistently shows that low mental health literacy significantly heightens the risk of adolescents developing moderate to severe depression. Enhancing mental health literacy may offer a valuable approach to reducing the future burden of common mental health problems among YP (10). School-based psychoeducational interventions are effective in reducing stigma, promoting YP’s mental health knowledge, and increasing mental health literacy but rely on educational engagement, leadership and delivery support at a local level (11). There is a dearth of co-produced and self-directed digital mental health literacy interventions for YP in the UK.

In the UK, school-based interventions to improve mental health literacy often include teacher-led psychoeducational sessions, such as the ‘MindEd’ programme or components embedded within the Personal, Social, Health and Economic education curriculum (12, 13). While these approaches have shown promise in improving knowledge and reducing stigma (14), they are typically not co-produced with young people and rely heavily on school staff capacity and willingness to deliver potentially sensitive material. There remains a gap for co-produced, self-guided digital interventions that can be used flexibly across educational and community settings, particularly those that harness the potential of technology to engage young people and support their mental health.

Digital applications have proven effective in managing mental health conditions such as anxiety, depression and stress, and offer a scalable solution to address the gap in co-produced, self-directed interventions aimed at improving mental health literacy among young people. Tools like cognitive behavioural therapy (CBT) apps, mindfulness platforms, and mood tracking systems have shown promising results. For example, a systematic review and meta-analysis found internet-based CBT as effective as face-to-face therapy (15). Similarly, mindfulness applications have been linked to reduced psychological distress and improved mental well-being (16). These findings underscore the scalability and efficacy of digital interventions in addressing mental health needs, particularly in underserved areas. However, their role in enhancing mental health literacy remains underexplored and warrants further investigation.

The Improving Mental Health Literacy Among Young People in Indonesia (IMPeTUs) intervention is a co-produced, evidence-based digital intervention designed to improve mental health literacy and develop self-management of anxiety and depression among young people aged 11–15 in Java, Indonesia (17). YP navigate through an immersive storyline that requires players to make decisions based on presented scenarios. The storylines, interspersed with mini-games designed to help players learn about positive mental health, simulate real-life scenarios where decisions can impact the direction and conclusion of the story. The IMPeTUs intervention has utilized these interactive elements to enhance mental health literacy and the self-management of anxiety and depression in YP in Indonesia, with levels of usability and acceptability (18).

With the number of YPS in the UK experiencing mental health problems increasing, and given that this demographic represents the peak age for depression onset and a high risk of recurrence throughout the lifespan, they must be equipped with knowledge and information regarding mental health, how to seek support, and strategies to cope with distress. Digital applications, proven successful in improving mental health and mental health literacy, are potentially cost-effective and practical methods to achieve this. The IMPeTUs intervention is a potential avenue to improve mental health literacy among YP in the UK, if modifications are made to reflect this demographic’s experiences accurately. The current study was conducted with the following aims:

1. To co-adapt an existing YP-centred digital application (the IMPeTUs intervention) for use in the UK.
2. To co-create additional digital application content with YP, professionals, parents, and community representatives.
3. To evaluate the feasibility of delivering the application in educational and community settings in the UK.
4. To evaluate the feasibility of undertaking a trial of the co-adapted IMPeTUs intervention in educational and community settings in the UK.

## 2 Materials and methods

### 2.1 Study design

The study consisted of two phases. Phase 1 focused on co-adapting a digital application to enhance mental health literacy in YP. Phase 2 aimed to assess the feasibility of trialling and implementing the application in community and educational settings in the UK with a mixed-method evaluation (19).

### 2.2 Phase 1: co-adaptation and co-design of the digital application

The original application, developed in Indonesia for YP aged 11–15 to enhance mental health literacy and self-management skills, was co-adapted and co-designed in this study for YP aged 12–15 in the UK. This process was guided by the principles of Experience-Based Co-Design (20) to ensure meaningful collaboration with stakeholders at each stage. The original IMPeTUs intervention was selected for adaptation due to its prior evidence of usability, narrative-based structure, and successful co-production in a non-Western context (17, 18), making it a strong candidate for culturally sensitive adaptation.

#### 2.2.1 Initial co-adaptation events

Phase 1 began with stakeholder consultation events designed to co-adapt existing content and co-design a new chapter based on priorities for UK YP. These events aimed to gather ideas on tailoring the application and identifying additional content needs. Stakeholders, invited via existing research contacts and outreach to community and educational sites in Greater Manchester, were recruited across five stakeholder groups: YP, health professionals, education professionals, parents of YP, and volunteer/community representatives. The research team presented the study and the original application to each group, followed by group discussions to collect feedback on the application’s content, design and functionality. The goal was to recruit 24–40 stakeholders for these events.

#### 2.2.2 Ongoing adaptation with advisory groups

Throughout the adaptation process, an Implementation Reference Group (IRG; n = 8), comprising health and educational professionals, and a Public Patient Involvement (PPI) advisory group (n = 6) consisting of YP with lived mental health experience, collaborated closely with the research team. Their role was to prioritize necessary adaptations to the application and provide overall study guidance and support. One PPI contributor actively participated in the IRG, co-adaptation and co-design events, and academic output, and was trained and supported to contribute to qualitative data analysis. The feedback from these groups was communicated to the application developers.

#### 2.2.3 Final review events

The final stage of Phase 1 involved reviewing the completed application with the stakeholder groups. Feedback from these events informed necessary adjustments before the feasibility trial. This phase also included finalizing instructions for downloading and operating the application, ensuring it was ready for use.

### 2.3 Phase 2: feasibility trial

Phase 2 consisted of a multi-site, cluster-randomized feasibility trial with a nested mixed-method process evaluation (19). The trial aimed to test the feasibility of delivering and evaluating the intervention in community and educational settings, focusing on recruitment, retention, and engagement metrics. While this intervention was designed as a universal approach, it was trialled only with interested, consenting individuals. Feedback from this population may be more positive than would be expected in routine implementation (21).

#### 2.3.1 Ethical approval and registration

Ethical approval was obtained from the University Research Ethics Committee (UREC 5: Ref 14361). The trial was prospectively registered with ISRCTN (ISRCTN16116467), detailing the study design, objectives, and outcome measures.

#### 2.3.2 Study sites and recruitment

The trial initially recruited four sites (two educational and two community settings) across Greater Manchester. Community settings included youth centres and voluntary organizations, ensuring a diverse range of non-health-focused contexts. Recruitment strategies included advertisements in school newsletters and direct invitations through site facilitators.

#### 2.3.3 Participants

**Figure 1:**
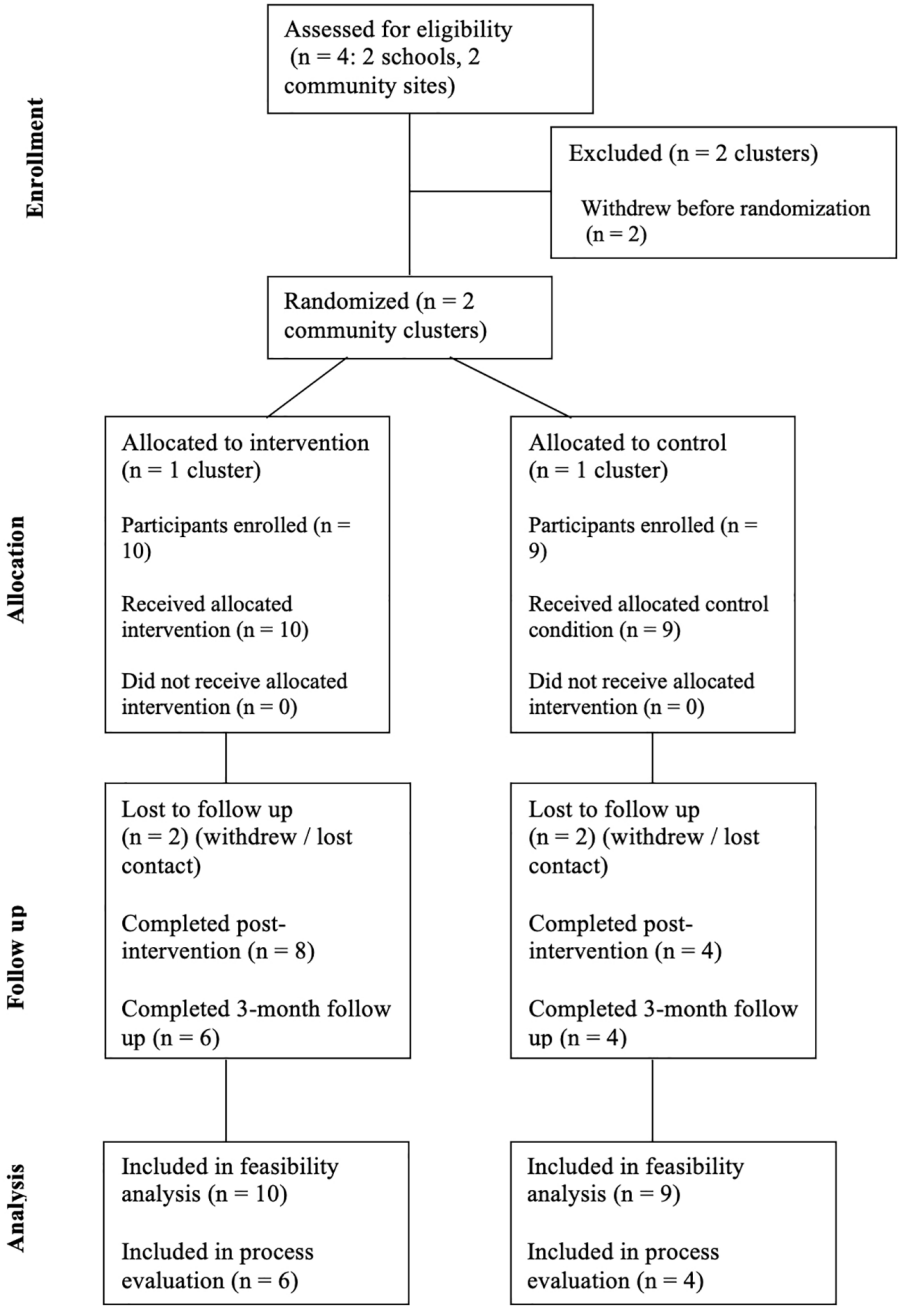
CONSORT diagram showing the flow of participants through each stage of the multi-site, cluster-randomized feasibility trial with nested mixed-methods.

Eligible participants were young people (YP) aged 12–14 who could provide assent, obtain parental consent, and have access to a smartphone or tablet. The original application, developed in Indonesia for YP aged 11–15, was co-adapted for YP aged 12–14 in the UK. This narrower age range was informed by feedback during the co-adaptation phase. UK YP and other stakeholders felt the original range was too broad for a developmentally focused intervention. A total of 19 YP aged 12–14 participated in the feasibility trial across two community sites. Of these, 10 YP completed all intervention components and participated in the process evaluation, including focus groups. Participants needed sufficient English proficiency and the capacity to engage with the intervention; see **Table 1** for full inclusion and exclusion criteria. Facilitators assessed the ability to engage with the app informally, based on young people’s access to a smartphone or tablet, English literacy, and ability to follow digital instructions independently. No participants were excluded based on their engagement ability. In addition, two parents/guardians and four facilitators participated in the process evaluation. See **Figure 1** for CONSORT diagram.

**Table 1:** Inclusion and exclusion criteria.

Inclusion criteria	Exclusion criteria
Aged 12–14 years	Younger than 12 or older than 14 years
Able to provide informed assent	Lacking capacity to provide informed assent
Parental consent obtained	Parental consent not obtained
Proficient in English	Insufficient English proficiency
Access to a smartphone or tablet	No access to required digital device
Capable of engaging with the intervention	Mental incapacity to participate

#### 2.3.4 Sample size justification

As this was a feasibility study, a formal power calculation was not required. However, a target of 40 participants was chosen based on the minimum needed to assess feasibility indicators and detect a standardised effect size of 0.25 in outcomes. This figure also allowed for anticipated attrition across the three study time points. Ultimately, 19 YP participated in the trial across the two study sites. While falling short of the target, this recruitment figure highlights potential barriers and informs planning for future trials. Given the small sample size, findings from this feasibility trial are indicative and not statistically conclusive.

#### 2.3.5 Randomization

Study sites were randomized into intervention and control arms using a block randomization list generated by the study statistician (JW). Allocation concealment was maintained until participant recruitment was complete. Control arm participants accessed the application after the study’s conclusion.

#### 2.3.6 Intervention

The intervention comprised the co-adapted digital application developed in Phase 1, supported by two facilitated group sessions.

The application featured three chapters focusing on key areas identified by stakeholders: (1) depression, (2) anxiety, (3) mental health and wellbeing. Each chapter used a narrative-based, choose-your-own-path format tailored to YP’s experiences in home, school, and social settings. To support skill-building, the application included embedded mini-games. One game resembled a search engine interface, where users were asked to evaluate the accuracy of online information related to emotional distress and symptoms of mental illness. Players selected the most appropriate responses to various scenarios, helping them practise identifying trustworthy sources and making informed decisions. Another mini-game introduced a grounding technique to promote calm during anxiety. Users were prompted to observe and name specific objects in their surroundings, such as “find something blue”, to develop mindfulness and sensory awareness. The application also included a distress button offering immediate access to external support resources. Young people accessed the application independently at home via their personal smartphones/tablets or on a computer at the host site.

Support was provided during group sessions in the community settings. These facilitated sessions introduced participants to the application, guided them on its use and encouraged discussion. Sessions took place before and after the four-week intervention period.

Based on the original IMPeTUs development, facilitation was key in intervention engagement, especially when delivered by a trusted individual in a ‘safe space’ setting (18). The minimum expected engagement included one hour per chapter and participation in both facilitated sessions, totalling approximately five to six hours.

#### 2.3.7 Procedures

Facilitators, nominated by participating sites, were trained to support recruitment, collect baseline data, and conduct group sessions. A total of six facilitators were initially trained: four from the community sites and two from the school sites. However, due to site withdrawals, only four facilitators from the community sites completed the process evaluation. Recruitment packs included age-appropriate materials for YP, information sheets for parents/guardians, and consent forms. Direct invitations and electronic consent forms enhanced accessibility for participants and their families. The process evaluation was guided by the Theoretical Framework of Acceptability (TFA), which examines seven dimensions of acceptability (22): affective attitude, burden, ethicality, intervention coherence, opportunity costs, perceived effectiveness, and self-efficacy. This framework informed the design of focus groups and interviews conducted with YP, parents/guardians, and facilitators, each lasting ~30 minutes, facilitated by HBrierley. For TFA component definitions, see **Table 2**.

**Table 2:** Theoretical framework of acceptability (TFA).

TFA component	Definition
Affective attitude	How participants feel about the application.
Burden	The effort participants feel they need to make to take part in the intervention.
Ethicality	The extent the intervention aligns with the values of the participant.
Intervention coherence	Whether participants understand how to use the intervention.
Opportunity costs	Whether any benefits, profits or values need to be sacrificed to take part in the intervention.
Perceived effectiveness	Whether participants felt the intervention operates with the intended purpose.
Self-efficacy	Whether participants feel they are confident in performing the behaviour(s) needed to take part in the intervention.

#### 2.3.8 Incentives and site support

Participants were reimbursed for their time: YP received £10 shopping vouchers for completing post-intervention questionnaires and 3-month follow-ups, with an additional £10 for process evaluation participation. Parents/guardians and facilitators received £25 shopping vouchers. Each study site received a one-time payment of £1000 for their involvement.

#### 2.3.9 Data collection

Phase 2 evaluated both implementation outcomes and exploratory effectiveness outcomes using quantitative and qualitative methods. The quantitative component treated feasibility indicators as primary outcomes, including recruitment, retention, and engagement rates. Measures of clinical change—mental health literacy, anxiety, depression, and well-being—were considered exploratory secondary outcomes.

The qualitative component used the TFA to explore implementation outcomes related to acceptability and usability. This included participants’ perceptions of the application and its usability, feedback on the study design, and suggestions for future improvement. Sections 2.3.9.1 and 2.3.9.2 provide full details of the quantitative and qualitative procedures, respectively.

##### 2.3.9.1 Quantitative data collection

Questionnaires were administered at three distinct time points: baseline (pre-randomization), post-intervention (~4 weeks post-randomization), and at a 3-month follow-up. These time points captured the intervention’s immediate and sustained impacts. YP in both the intervention and control arms completed the questionnaires.

###### 2.3.9.1.1 Primary outcomes

The primary outcomes focused on feasibility metrics, including recruitment and retention rates, intervention uptake, and engagement rates.

###### 2.3.9.1.2 Exploratory secondary outcomes

Secondary outcomes included the variability and potential floor/ceiling effects of proposed clinical measures and the assessment of mental health literacy, anxiety, depression, and overall well-being.

###### 2.3.9.1.3 Questionnaires

The selected measures, summarized in **Table 3**, were based on expert consultation and aimed to understand the intervention’s impact comprehensively.

**Table 3:** Measures and timepoints.

Outcome measure	Purpose	Time points
Demographics and psychiatric history questionnaire	Purpose developed for the study to collect participant characteristics	Baseline
Knowledge and Attitudes to Mental Health Scale (KAMHS)	Primary clinical outcome, assesses mental health literacy	Baseline, post intervention, 3 month follow up
Mood and Feeling Questionnaire (MFQ)	Measure depressive symptoms	Baseline, post intervention, 3 month follow up
World Health Organization Five Well-Being Index (WHO-5)	Assess subjective wellbeing	Baseline, post intervention, 3 month follow up
Revised Children’s Anxiety and Depression Scale (RCADS)	Measure anxiety and depression symptoms	Baseline, post intervention, 3 month follow up
Family Adaptability and Cohesion Evaluation Scale (FACESIV)- the Family Cohesion and Satisfaction with Communication sub-scales	Assess family communication and cohesion	Baseline, post intervention, 3 month follow up
SF-36 quality of life questionnaire	Measure overall quality of life	Baseline, post intervention, 3 month follow up
Intervention engagement questionnaire	Assess engagement with the intervention	Baseline, post intervention, 3 month follow up

All questionnaires used have demonstrated acceptable reliability and validity in adolescent populations. The Mood and Feelings Questionnaire (MFQ), which assesses depressive symptoms, is a unidimensional scale with Cronbach’s alpha values typically exceeding 0.90 in adolescent samples (23). The Revised Child Anxiety and Depression Scale (RCADS) includes multiple subscales for anxiety and depression, each of which has demonstrated acceptable internal consistency (α = 0.70–0.85) and strong construct validity in both clinical and community youth samples (24). The WHO-5 Well-Being Index is a brief, unidimensional measure of subjective well-being with reported alpha values around 0.85–0.90 in adolescent populations (25). The FACES-IV subscales used in this study (Communication and Satisfaction) demonstrated good reliability in adolescent samples (26). Finally, the SF-36, though originally developed for adult populations, has been validated for adolescent use, with acceptable reliability reported across its eight physical and mental health domains (27).

##### 2.3.9.2 Qualitative data collection

After approximately six weeks of accessing the application, YP in the intervention arm were invited to participate in a one-to-one interview with the researcher or a focus group with other YP from their study site, depending on their preference. All YP opted to join a 30-minute focus group facilitated by one of their site facilitators and a research team member.

The focus group utilized a topic guide informed by the TFA, exploring YP perceptions of the application and its usability; feedback on the study design; suggestions for future improvements.

Similar topic guides, also informed by the TFA, were used for focus groups and interviews with parents/guardians and facilitators to gain insight into their perspectives on YP’s experiences with the application. These sessions were conducted via Zoom, digitally recorded, and transcribed verbatim.

#### 2.3.10 Data analysis

##### 2.3.10.1 Quantitative analysis

Quantitative data analysis was primarily descriptive and focused on determining the feasibility of the study. The analysis included summarizing the proportion of missing data for each variable and comparing means at each follow-up time point using ANCOVA, adjusting for the corresponding baseline measures. As exploratory analyses, 95% confidence intervals were reported, but p-values were not included, as the focus of the study was feasibility rather than hypothesis testing.

Feasibility outcomes were assessed using a traffic light system, with criteria pre-defined by the research team, as shown in **Table 4**. This system provided thresholds for evaluating recruitment, retention, and engagement metrics. Summaries of questionnaire scores are presented in **Table 5**. Descriptive statistics were used to assess the completeness and variability of participant and cost outcome measures. At baseline, an agreement scale was used for satisfaction questions instead of the satisfaction subscale from FACES-IV. Since both scales employed a 1–5 range, this difference is unlikely to impact the results significantly.

**Table 4:** Feasibility outcome criteria.

Criteria	Assessment			Additional information
	Red	Amber	Green	
Willingness of participants to be randomized	Recruited <60%	Recruited 60-80%	Recruited >80%	Based on the required sample size (40 participants).
Retention in intervention arm	Retained <60%	Retained 60-80%	Retained >80%	Based on retention at the 3-month follow-up.
Uptake of intervention	Engaged <60%	Engaged 60-80%	Engaged >80%	Minimum of one-hour engagement with each chapter and attendance of both facilitated sessions (five-six hours total).

**Table 5:** Modifications to application.

Participant	What needed to be modified	Why did it need to be modified	How did it need to be modified
YP	Delivery of information	Too much text to read	Add a voiceover option
		Text might be hard to read	Bigger font size
	Design of the application	Increase engagement	More customisation options: MC (clothing, hairstyles) Storyline backgrounds/rooms
			Add more avatars (e.g. siblings)
	Format of the application	To provide more helpful, personalised information	Be able to input own conditions into application to target content
		To get access to information without going into a Book	Have a separate resource page
Facilitators	Design of the application	Increase engagement	Include music (e.g. calming music, appropriate for those with ADHD)
			Brighter colours
			Include ‘current’ characters popular with YP
			Include animation, make interactive
	Format of the application	Increase engagement	Allow YP to connect with other YP in the application
Parents/guardians	Delivery of information	Too much text to read	Add voiceover option
	Design of the application	Increase engagement	More customisation options: Storyline backgrounds (colour) Voice options MC/characters
			Include animation, make interactive (e.g. pop ups, click options)
	Format of the application	Increase engagement	Add award system to indicate completion (e.g. certificates)
			Add quizzes to consolidate learning
			Include non-mental health content (e.g. interesting titbits/quotes)
			Add notification option (e.g. to get moving- connect to physical health)
		To provide personalised feedback	Be able to input in own details (e.g. feelings) to target content (e.g. games which will provide YP with personalised feedback)

##### 2.3.10.2 Qualitative analysis

The qualitative data were analysed using a framework approach that combined deductive and inductive coding (28). Deductive coding was guided by the Theoretical Framework of Acceptability (TFA), while inductive coding captured additional relevant themes outside the TFA framework.

After transcription and anonymisation of transcripts, multiple researchers participated in coding to ensure reliability and depth of analysis. HBrierley coded all transcripts, while NU double-coded two transcripts for reliability purposes. EBee also reviewed a selection of transcripts, adding further perspectives and ensuring that the coding process captured all relevant themes. Researchers familiarized themselves with the data through active reading of the transcripts.

The data were then charted into a pre-existing matrix based on TFA components, with written summaries and supporting quotes for each transcript. This matrix also allowed the inclusion of additional themes relevant to intervention analysis. Following the analysis, three authors (HBrooks, HBrierley, and NU) reviewed and confirmed the charted data. Their discussions focused on identifying factors influencing intervention acceptability. Detailed write-ups were subsequently prepared for each TFA component, accompanied by comments on other aspects of the study and suggestions for improving the application’s future usability.

## 3 Results

### 3.1 Participant characteristics

A total of 19 YP were involved in the trial across the two community sites. Of these, 10 YP completed all intervention parts, including focus groups, and participated in the process evaluation. Additionally, two parents/guardians and four facilitators took part in the process evaluation. Although six facilitators were initially trained, including two from school sites that later withdrew, only four facilitators from the community sites completed the process evaluation. Baseline demographic information and baseline outcome measure scores are presented in **Table 6**.

**Table 6:** Demographics and baseline outcome measurements of YP participants.

Demographics of participants (N = 19) *(n, % of participants in the total number of participants)*	Treatment *(n=11)*	Control *(n=8)*
Gender		
Female (17, 89%)	11 (100%)	6 (75%)
Male (2, 11%)	0 (0%)	2 (25%)
Age in years		
12 (8, 42%)	6 (54.5%)	2 (25%)
13 (9, 47%)	5 (45.5%)	4 (50%)
14 (2, 11%)	0 (0%)	2 (25%)
Ethnicity		
White (14, 74%)	6 (54.5%)	8 (100%)
Black (0, 0%)	0 (0%)	0 (0%)
Mixed ethnicity (3, 16%)	3 (27.3%)	0 (0%)
Other (2, 11%)	2 (18.2%)	0 (0%)
Past experience of mental health problems		
Yes (0, 0%)	0 (0%)	0 (0%)
No (13, 68%)	11 (100%)	2 (25%)
Don’t know (6, 32%)	0 (0%)	6 (75%)
Questionnaires (Baseline Outcome Measurements)		
KAHMS	22.17 (0.74) *0%*	21.04 (0.95) *0%*
WHO	24.18 (0.87) *0%*	12.00 (4.60) *0%*
MFQ	0.18 (0.60) *0%*	10.25 (8.03) *0%*
FACES		
Communication	37.91 (0.94) *0%*	35.25 (9.36) *50%*
Satisfaction	40.64 (0.67) *0%*	33.67 (10.02) *62.5%*
SF36		
Physical functioning	100 (0) *0%*	100 (0) *0%*
Role limitations due to physical health	100 (0) *0%*	100 (0) *25%*
Role limitations due to emotional problems	100 (0) *0%*	100 (0) *25%*
Emotional wellbeing	100 (0) *0%*	100 (0) *50%*
Energy/Fatigue	96.97 (10.05) *0%*	100.00 (NA)
Social functioning	NA *100%*	*87.5%*
Pain	NA *100%*	100 (0) *50%*
General Health	100 (0) *0%*	NA *100%* 100 (NA) *87.5%*
RCADS		
Major depression disorder	30.70 (2.21) *9.1%*	56.12 (16.36) *0%*
Obsessive compulsive disorder	35.00 (0.00) *0%*	51.00 (15.78) *0%*
Social phobia	25.20 (2.70) *9.1%*	50.88 (17.53) *0%*
Separation anxiety disorder	39.40 (1.26) *9.1%*	56.0 (12.93) *0%*
Pain disorder	37.27 (0.90) *0%*	60.50 (16.39) *0%*
General anxiety disorder	27.55 (1.81) *0%*	47.75 (13.60) *0%*

*N.B.* Mean score (SD). Missing (%) shown in *italics* for Questionnaires.

### 3.2 Phase 1: co-adaptation of the digital application

A total of 49 stakeholders participated in the consultation events, surpassing the initial target of 24–40 stakeholders. These included 14 YP, eight health professionals, nine education professionals, 11 parents, and seven voluntary and community representatives.

#### 3.2.1 Modifications to the application

The digital intervention was updated to enhance inclusivity, usability, and relevance for young people in the UK. Key enhancements included expanded character customisation (e.g., gender-neutral options, diverse physical attributes), UK-specific settings, and a new chapter addressing resilience, healthy relationships, and self-care. Stakeholder feedback, including input from young people, parents, and facilitators, highlighted the need for improved engagement and accessibility, such as reducing text, adding voiceovers, and introducing interactive features. Recommendations and their rationale are outlined in **Table 5**, including suggestions for progress-tracking systems and personalised content to enhance the user experience. The **Supplementary Materials** detail these recommendations’ prioritisation and implementation status (**Supplementary Tables 1-2**) and provide screenshots of the finalised application (**Supplementary Figures 1-4**). While some features, such as voiceovers and animations, were deferred due to funding constraints, they remain priorities for future development. This iterative process has informed ongoing improvements and highlighted areas for further refinement.

### 3.3 Phase 2: feasibility of trial and delivering intervention

#### 3.3.1 Quantitative results

Nineteen YP consented to be involved in the study and provided baseline and demographic data. Of these, 12 participants provided post-intervention data, and 11 participants provided data at the three-month follow-up. Those who dropped out no longer wished to provide further data. All assessments, including partially completed ones, were included in the analysis. Most participants were female (89%), aged 13 (47%), white (74%), and had no past experience of mental health problems (68%), as shown in **Table 6**.

##### 3.3.1.1 Recruitment and willingness to be randomized

Across the two participating community sites, recruitment efforts were successful at a site level, with Community Site 1 recruiting 11 YP and Community Site 2 recruiting 8 YP. However, the overall recruitment fell below 60% of the planned target of 40 participants, resulting in a Red outcome for recruitment based on the traffic light criteria. Despite this, the study demonstrated the feasibility of recruitment within community sites, despite challenges with school site recruitment.

One participant from Community Site 1 withdrew from the study before the first facilitated group session. All remaining participants completed baseline data collection before site randomisation.

##### 3.3.1.2 Retention in the intervention arm

Of the 10 YP initially enrolled in the intervention arm at Community Site 1, seven participants (70%) completed the post-intervention questionnaire, and five participants (50%) completed the three-month follow-up questionnaire. At Community Site 2, of the eight YP in the control arm, five participants (63%) completed the post-intervention questionnaire, and six (75%) completed the three-month follow-up questionnaire. Based on the traffic light criteria, retention at three months in the intervention arm was rated Amber (60–80%). However, retention at Community Site 1 (50%) was below this threshold, highlighting site variability. Given the small sample size, feasibility findings should be interpreted cautiously and are not generalisable.

##### 3.3.1.3 Uptake of the intervention

Among the 11 YP recruited at Community Site 1, 10 participants (91%) engaged with the intervention at the requisite level, completing at least one hour of engagement with each chapter and attending the facilitated group sessions. This resulted in a Green rating for intervention engagement. Group sessions supported intervention delivery in this feasibility study; their inclusion in future implementations is recommended but may vary depending on the setting and available resources. While engagement data indicated strong participation in the intervention, additional details on individual module usage and time spent within the application could enhance future analyses and refinement of the intervention.

##### 3.3.1.4 Exploratory analysis of outcomes

The inability to proceed with the four planned study sites resulted in a much lower sample size than anticipated. This limitation restricted the scope of the planned exploratory inferential analyses. Therefore, care should be taken when interpreting the results, as the small sample size limits the generalizability of the findings.

The exploratory analyses focused on examining trends in clinical outcomes based on questionnaire data, as detailed in **Table 7**. While some descriptive trends suggested potential benefits of the intervention, confidence intervals for most adjusted mean differences crossed zero, indicating no statistically robust differences between the intervention and control arms. Further research with a larger sample size is necessary to draw more definitive conclusions about the intervention’s effectiveness.

**Table 7:** Descriptive and inferential statistics of each questionnaire score according to their domains and time points.

Variable	Treatment *(n=11)*	Control *(n=8)*	Adjusted mean difference [95% CI]*
KAMHS			
1 month	21.10 (1.34) *36.4%*	20.51 (1.46) *37.5%*	-0.27 [-1.32;0.78]
3 month	21.84 (1.56) *54.5%*	20.48 (1.52) *25%*	0.71 [-0.71;2.12]
WHO			
1 month	15.33 (6.09) *45.5%*	12.80 (8.50) *37.5%*	-14.29 [-39.96;11.38]
3 month	17.50 (6.56) *63.6%*	12.67 (4.27) *25%*	-7.24 [-21.80;7.32]
MFQ			
1 month	10.80 (8.41) *54.5%*	11.00 (9.87) *37.5%*	9.05 [-4.38;22.49]
3 month	3.60 (6.07) *54.5%*	12.67 (7.81) *25%*	-0.55 [-10.63;9.53]
FACES Communication			
1 month	42.50 (4.81) *45.5%*	39.00 (9.95) *37.5%*	3.48 [-3.83;10;80]
3 month	42.00 (4.08) *63.6%*	36.00 (7.62) *25%*	2.78 [-6.01;11.6]
FACES Satisfaction			
1 month	38.50 (3.39) *45.5%*	36.80 (9.88) *37.5%*	0.18 [-8.10;8.46]
3 month	40.25 (8.18) *63.6%*	29.67 (10.44) *25%*	1.41 [-18.44;21.27]
SF36 Physical functioning			
1 month	100 (0) *54.5%*	100 (0) *37.5%*	NE
3 month	100 (0) *63.6%*	100 (0) *25%*	NE
SF36 Role limitations due to physical health			
1 month	100 (0) *45.5%*	100 (0) *50%*	NE
3 month	100 (0) *63.6%*	100 (0) *50%*	NE
SF36 Role limitations due to emotional problems			
1 month	100 (0) *54.5%*	100 (0) *37.5%*	NE
3 month	100 (0) *63.6%*	100 (0) *50%*	NE
SF36 Emotional well-being			
1 month	100.00 (0) *45.5%*	100.00 (0) *62.5%*	NE
3 month	100.00 (0) *63.6%*	100.00 (0) *75%*	NE
SF36 Energy/Fatigue			
1 month	72.22 (25.46) *72.7%*	100.00 (0) *75%*	-25.00 [-16.26;16.76]
3 month	100.00 (0) *81.8%*	100 (NA) *87.5%*	NE
SF36 Social functioning			
1 month	100.00 (0) *63.6%*	100.00 (0) *75%*	NE
3 month	100.00 (0) *63.6%*	100.00 (0) *62.5%*	NE
SF36 Pain			
1 month	NA *100%*	NA *100%*	NE
3 month	NA *100%*	NA *100%*	NE
SF36 General health			
1 month	100 (0) *63.6%*	100 (0) *62.5%*	NE
3 month	100 (0) *72.7%*	100 (0) *75%*	NE
RCADS Major depressive disorder			
1 month	52.14 (22.15) *36.4%*	52.20 (12.64) *37.5%*	10.94 [-34.53;56.40]
3 month	33.00 (3.56) *63.6%*	62.5 (17.21) *25%*	-6.67 [-25.16;11.83]
RCADS Obsessive compulsive disorder			
1 month	42.71 (9.93) *36.4%*	47.40 (6.99) *37.5%*	-0.30 [-15.25;14.66]
3 month	35.75 (1.50) *63.6%*	52.83 (11.86) *25%*	-8.38 [-22.05;5.28]
RCADS Social phobia			
1 month	45.14 (12.95) *36.4%*	59.40 (17.74) *37.5%*	2.49 [-26.02;31.10]
3 month	38.00 (12.11) *63.6%*	58.17 (17.37) *25%*	0.68 [-18.88;20.23]
RCADS Separation anxiety disorder			
1 month	49.99 (11.94) *36.4%*	66.60 (19.58) *37.5%*	-6.93 [-48.00;34.15]
3 month	42.75 (2.87) *63.6%*	73.83 (26.97) *25%*	-5.86 [-64.88;53.17]
RCADS Panic disorder			
1 month	49.14 (17.99) *36.3%*	58.40 (12.30) *37.5%*	6.08 [-26.29;38.46]
3 month	42.25 (3.77) *63.6%*	65.33 (14.77) *25%*	-1.23 [-14.20;11.68]
RCADS General anxiety disorder			
1 month	44.57 (14.33) 36.3%	56.00 (18.33) *37.5%*	15.39 [-17.48;48.26]
3 month	39.50 (19.16) *63.6%*	57.33 (15.85) *25%*	8.83 [-27.56;45.22]

*N.B.* Mean (S.D) % Missingness in *italics*, *ANCOVA adjusted for baseline value, NE; Not estimable.

#### 3.3.2 Qualitative results

The process evaluation involved YP (n = 11), facilitators (n = 4), and parents (n = 2). Feedback from these participants is presented below under the relevant TFA subheading (see **Table 3**). After the study, other young people (YP) were also given access to the application if they expressed interest.

##### 3.3.2.1 Overall impressions

Participants from all three stakeholder groups found the application’s content helpful and informative, but they identified areas where the format could be improved to enhance engagement. Specifically, the application was described as too text-heavy, with participants suggesting that its narrative and reading elements should be streamlined to make it more accessible.

Initially, both facilitators and parents expressed uncertainty about what to expect from the application. Facilitators recognized that the application met its intended purpose of improving mental health literacy in YP, despite initial ambiguity about its format.

“I didn’t know what I was expecting [ … ] but, no, I think the expectation has been met in terms of you know, obviously it was designed for a purpose” [Facilitator 1]

Parents anticipated a more game-like experience and noted that the application was more story-oriented than expected. This mismatch in expectations highlighted the importance of marketing the application to align with its narrative-based structure. One parent noted that the app was educational rather than game-like, influencing their overall impression of its utility.

“I wouldn’t even class it as a game. When I went on it, to me, it wasn’t a game. It was more … it was educational, that’s for sure.” [Parent 1]

YP echoed this sentiment, with one participant commenting that the application required *“a lot of reading” [YP 1].* While YP found the content relatable and helpful, the reading-heavy nature posed challenges for engagement, particularly for those less comfortable with or interested in reading.

##### 3.3.2.2 Burden

Both YP and facilitators encountered initial challenges when downloading the application onto devices. The most significant burden for YP was the excessive reading required to progress through the storylines. This was particularly difficult for YP, who struggled with or disliked reading, as they focused more on completing the text than engaging with the storyline’s content.

“People who may struggle with reading or don’t really enjoy it as much [ … ] might find it harder to concentrate on the information and the story, and they might be concentrating on getting through all the writing” [YP 1]

Parents also found the application time-consuming, expressing concerns that the lengthy storylines might reduce patience and lead to missed information. These observations underscore the need to ensure accessibility by reducing text, adding voiceover options, or introducing other features to maintain engagement without compromising the educational value.

##### 3.3.2.3 Affective attitude

Both facilitators and YP enjoyed the digital application and facilitated group sessions. There was consensus that the group sessions were beneficial, providing an opportunity for YP to talk with each other in a supportive environment. However, it was noted that some YP might need more encouragement than others to be vocal in such a setting, highlighting the need for experienced facilitators.

When considering the application, YP particularly appreciated the relatability of the storylines and the mini-games interspersed throughout the stories. They found the application helpful as it demonstrated that there can be multiple solutions to individual and isolated problems. YP felt that the application shows that it is not always a case of one problem and one solution, but that there are several potentially helpful ways to address YP’s challenges. Facilitators further emphasised the importance of YP being presented with various “solutions” provided in the application.

“I really liked how there were loads of solutions to some problems” [YP 2]

Facilitators also appreciated how the application encouraged YP to consider not only their mental health but also the mental health of those around them. This suggests that the application helped YP foster a sense of awareness towards the well-being of others.

One downside of the application was the desire YP expressed for more customisation options, further highlighting this demographic’s importance on relatability. Additionally, YP noted that the application required a lot of reading. Parents echoed these sentiments, mentioning that the dialogue-heavy storylines were too long and could be overwhelming. This feedback further underscores the importance of highlighting the narrative-heavy aspect of the application when marketing to YP. It also introduces the necessity of making the storyline more accessible by reducing the amount of reading required, all while ensuring the content remains impactful for improving mental health literacy among YP.

“I think there could be a smidge more options of clothing and hairstyles” [YP 3]

##### 3.3.2.4 Ethicality

Feedback from both facilitators and parents indicates that integrating the application into the lives of YP could be seamless. There was a suggestion that YP are already accustomed to using educational and mental health-related applications, facilitating easy adoption of this application into their existing routines.

“Everyone spends so much time on devices and they don’t have time for much else [ … ] so if you can move the education on to the device, and they are quite used to playing other games. Like they use an app called Seneca, which is a science app. So they are quite used to using that for school. So this, kind of, fits along that” [Parent 2]

Furthermore, integrating the application into existing activities conducted by community organisations and schools was proposed as a viable approach. Facilitators emphasised that providing access to the application would be beneficial for YP, especially considering the current challenges they face in accessing additional mental health support. This integration holds the potential to enhance the overall accessibility and effectiveness of the application in supporting YP’s mental health needs. Additionally, facilitators liked the distress button feature, which provided YP with resources for further support. Parents expressed the importance of ensuring their children’s safety using the application. They appreciated having *“peace of mind” [Parent 1]* knowing that strangers could not contact YP through the application. This reassurance made parents comfortable, allowing YP to use the application without supervision.

##### 3.3.2.5 Intervention coherence

YP and their parents found the application intuitive and straightforward to use. One parent noted that their child had no issues navigating the application independently, highlighting its user-friendly design.

“She [YP] thought it was quite easy to use. She liked the layout [ … ]” [Parent 2]

Despite its usability, the text-heavy nature of the application limited its accessibility for some YP, reinforcing the need for adjustments to ensure broader engagement.

##### 3.3.2.6 Opportunity costs

Based on the comments provided, the primary cost associated with using the application was the significant amount of time required. Parents expressed concerns that this could detract from other activities, such as completing homework or playing other digital games.

“She’s [YP] probably spent about three or four hours on it going through things. She has a lot of homework, and she did go through the Zoom meeting [ … ] I don’t know how often she played it but it was about, I think she spent about four hours doing it in total” [Parent 2]

##### 3.3.2.7 Perceived effectiveness

Feedback from YP, facilitators, and parents highlights the utility of the application’s content. YP valued the quality of the resources included in the application and appreciated that they had been “vetted” by reliable individuals. The term “vetted” refers to the careful selection and review of resources by mental health professionals to ensure they are accurate, age-appropriate, and free from potentially harmful content. This aspect was considered crucial, as the application provided a safer alternative than YP independently searching for information online, where they could encounter harmful content. Facilitators and parents supported using the application for this purpose, recognising its role in providing YP with reliable and safe information on mental health topics. They viewed the app as a beneficial alternative for YP who may not feel comfortable contacting others for support.

“If you can’t talk to your mum or you can’t talk to a supervised adult, then this is an app where you can go and get the information that you need and the help and support that you may require” [Parent 1]

However, concerns were raised about introducing mental health topics to YP who have limited prior knowledge, as this could potentially overwhelm them. Therefore, there was consensus on the importance of supporting YP who may find the content challenging or distressing. This approach would ensure that the application serves as a supportive tool in fostering mental health literacy while maintaining sensitivity to the individual needs of YP.

##### 3.3.2.8 Self-efficacy

Based on the comments made by YP, facilitators and parents, coupled with the absence of identified usability issues, YP demonstrated confidence in using the application. This indicates that the application effectively met user expectations and was intuitive in its design and functionality, contributing to a positive user experience among YP.

##### 3.3.2.9 Other study aspects

During the process evaluation, we also asked questions about recruitment, the questionnaires and training provided and improvements that should be made to the study.

##### 3.3.2.10 Recruitment

Recruiting study sites for the application proved challenging, particularly with schools withdrawing and difficulties in recruiting YP at community sites. Facilitators highlighted that engaging with school wellbeing officers early could be a pivotal strategy for improving recruitment in future studies. Wellbeing officers, who often serve as key contacts for student support, could act as vital gateways to facilitating access to schools and their YP populations.

“If anything, maybe getting connected with the schools’ wellbeing officers. They may be that gateway through [to recruiting school sites].” [Facilitator 2]

At one community site, which did not directly engage with YP, recruitment efforts relied on personal networks, such as friends, neighbours, and family members associated with the organisation. Conversely, another community site faced challenges with YP’s voluntary and inconsistent attendance and limited contact with parents, which hindered obtaining consent. These challenges further underscore the importance of early engagement with schools, particularly through wellbeing officers, to establish clear timelines and improve recruitment success.

##### 3.3.2.11 Questionnaires

Facilitators praised the content thoroughness of the questionnaires, and both the YP and parents affirmed their clarity. However, there was a consensus that the questionnaires contained too many questions, which YP found overwhelming, especially since many had not previously encountered such extensive surveys. At one community site, encouraging YP to complete the questionnaires disrupted their activities and diverted facilitators from their regular duties. Facilitators proposed breaking down the questionnaires into smaller segments or scheduling targeted sessions where all YP could complete them simultaneously, though confidentiality concerns were noted. Alternatively, some parents suggested that YP might prefer group discussions over questionnaires.

“That [the questionnaire] was the bit, the lengthy bit of it and if they’ve not been in situations like that [filling in exhaustive questionnaires] or have had experiences of topics like that, they could feel quite overwhelmed. ‘Why am I filling this in?’” [Facilitator 2]

All agreed that facilitator training was adequate, although one facilitator expressed a desire for earlier involvement in the study to provide feedback on the application before implementation.

##### 3.3.2.12 Other study improvements

In addition to the suggestions for improving recruitment and data collection previously discussed, another recommendation was to expand the application’s target age range to include 10–17-year-olds, thereby broadening its reach among YP.

“I would [extend the age range] because sometimes you get a 16-year-old or a 15 or a 17-year-old [look for support]” [Facilitator 1]

While there were initial reasons for narrowing the age range, this suggestion highlights a potential future consideration to maximise the application’s impact across a wider age group. This adjustment could enhance accessibility and relevance, accommodating a broader spectrum of YP who could benefit from the application’s resources and support for mental health literacy.

## 4 Discussion

The primary aim of this study was to co-produce a digital application tailored to the needs of YP in the UK and to evaluate the feasibility of implementing and assessing its use in a trial setting. The study involved 49 stakeholders, including YP, parents, facilitators, PPI contributors, and members of an IRG, whose diverse input informed the development and adaptation of the application. Phase 1 was highly successful, with stakeholder collaboration resulting in a well-received application that reflected the needs and preferences of its target audience. This participatory approach provided valuable insights into the intervention’s design and the practicalities of conducting a feasibility trial and has been recognized in the literature as a critical factor in enhancing user engagement and satisfaction while improving the relevance and acceptability of interventions (29). The application developed in this study, based on the Indonesian IMPeTUs model, was positively received by YP. Participants’ feedback indicated that while the core content and usability of the application were appropriate, further modifications are required to enhance engagement and accessibility. These findings align with previous research, highlighting the effectiveness of co-produced digital mental health interventions in increasing mental health literacy among young users (18, 30, 31). Given the small sample size and self-selecting nature of participants, findings from this feasibility study are indicative only and should not be generalised beyond the study context.

However, Phase 2 presented significant challenges. Recruitment and retention issues, particularly the dropout of school sites, meant that the target sample size was not achieved. While community sites successfully recruited YP, voluntary attendance and limited parental contact created barriers to consistent participation. These recruitment difficulties highlight the need for improved contextual integration, as evidenced by challenges embedding the intervention into existing school workflows. The Normalisation Process Model (32) provides a valuable framework for understanding these barriers as systemic issues in incorporating new interventions into institutional practices. Suggestions to address these challenges include engaging school wellbeing officers early in the planning process, establishing clear timelines, and consulting researchers with expertise in school-based studies. Wellbeing officers, in particular, are ideally positioned to act as gateways to accessing YP populations and supporting trial implementation. These strategies are consistent with research emphasizing the importance of strong partnerships with educational institutions and community organizations for successful school-based research (33).

The volume of questionnaires administered during the study also presented a challenge. While the questionnaires were not unduly long compared to other core outcomes used in trials, some YP found the volume overwhelming, particularly those unaccustomed to completing detailed surveys. This highlights the need to further consider the balance between participant burden and the required data quality. Such an approach would ensure that the information collected is valuable for educational and health policy decisions without compromising user engagement. Previous research has shown that overly long questionnaires can lead to participant fatigue and diminished data quality, underscoring the importance of carefully designing these measures (34).

Feedback from participants suggested a range of modifications to improve the application’s accessibility and engagement. This feedback was interpreted through the Theoretical Framework of Acceptability (TFA), which helped identify sources of burden, intervention coherence, and perceived effectiveness from participants’ perspectives. One of the most significant issues raised was the text-heavy nature of the application, which posed challenges for YP with lower reading abilities or limited patience for lengthy narratives. Reducing text and introducing voiceovers were suggested practical solutions to address these barriers. Participants also recommended incorporating interactive elements, such as quizzes, animations, or customization features, to make the application more engaging. These enhancements could help the application stand out in a crowded market of game-like mental health apps while maintaining its unique focus on education and mental health literacy. Although the application is not designed to compete directly with game-like apps, offering engaging features could increase the likelihood of YP choosing and using it consistently. These findings align with studies demonstrating that digital applications are most effective when tailored to their target audience and designed to foster engagement through interactive elements (15, 16).

Despite these challenges, the application’s content was well-received. Facilitators and parents valued the inclusion of vetted mental health resources, which provided a safe and reliable alternative to unsupervised online searches. The application ensured users could access accurate and age-appropriate information by offering pre-approved resources tailored to YP’s needs. However, introducing mental health topics to YP with limited prior knowledge must be approached carefully to avoid overwhelming users. Stakeholders highlighted the importance of supporting YP who may find some content challenging or distressing, ensuring that the application continues to serve as a supportive tool for fostering mental health literacy. While group sessions supported the intervention in this feasibility study, the application was designed for self-directed use. Future implementation should still utilise group facilitators, as the current study and previous findings (18) demonstrate that facilitation was key to engagement.

Several recommendations emerged from this study that are crucial for designing and implementing future trials. Recruitment strategies should prioritize the involvement of school wellbeing officers, as they play a critical role in facilitating access to YP populations and supporting schools through the trial process. Co-selecting questionnaire measures with YP and school staff could strike an optimal balance between minimizing participant burden and collecting data valuable for policy and commissioning decisions. Future iterations of the application should focus on improving accessibility by addressing text-heavy elements, incorporating voiceovers, and introducing more interactive features to enhance engagement. Although a broader age range (e.g. 10–17) was discussed during the process evaluation, any expansion would require further co-design and piloting to ensure developmental appropriateness.

The involvement of PPI contributors and an IRG was a strength of this study, as it ensured that the application was designed with input from those intended to benefit. However, the challenges identified, particularly in recruitment and questionnaire burden, underscore the need for further refinement of trial processes to support large-scale evaluations. Addressing these challenges through improved recruitment strategies, co-designed measures, and targeted application enhancements will be essential for the success of future trials. Securing sufficient funding and resources to implement these strategies will also play a critical role. This study provides valuable insights into the feasibility of implementing a digital application for YP’s mental health literacy, offering a strong foundation for the continued development and evaluation of such interventions.

### 4.1 Future work

Comments during the process evaluation highlighted several potential modifications to improve engagement. Many of these suggestions mirrored feedback received from stakeholders during Phase 1 of the study, but could not be implemented due to time and budget constraints. Participants expressed a preference for more game-like elements in the application, such as interactive features and customization options. While the application is not designed to compete directly with other game-like apps in this space, incorporating these modifications could increase its appeal and enhance its likelihood of being chosen and used by YP. Striking the right balance between educational content and engaging features is crucial to ensuring that the application remains unique in its focus on improving mental health literacy while meeting user expectations for accessibility and entertainment.

The application evaluation method faced several challenges, particularly regarding school recruitment and implementation. Facilitators noted that aspects of the process were time-consuming and challenging to integrate alongside their existing roles. This lack of contextual integration may have contributed to the withdrawal of initially recruited schools. Using the Normalisation Process Model (24) as a framework, these difficulties can be understood as barriers to embedding the intervention into existing school workflows and routines. To address these issues, suggestions included engaging school wellbeing officers early in the process, establishing clear timelines, and consulting researchers with experience in school-based studies to better understand and mitigate recruitment challenges. By improving contextual integration, future studies may achieve greater success in school recruitment and retention.

### 4.2 Conclusion

In conclusion, while the digital application demonstrated potential for improving mental health literacy, further work is needed to refine its design and format to enhance engagement. Additionally, future studies should consider alternative methods to reduce the burden on study sites and participants, thereby minimising dropout rates and improving overall feasibility. Engaging with school wellbeing officers and consulting with researchers experienced in school-based studies could provide valuable insights into overcoming recruitment challenges. Overall, this study contributes early insights into the iterative development and evaluation of digital mental health interventions for YP. Further research is needed to establish acceptability and effectiveness in larger, more diverse samples.

## Data Availability

The raw data supporting the conclusions of this article will be made available by the authors, without undue reservation.
