# Supplementary material for: Bridging the gap in mental health literacy: co-adapting and feasibility testing a digital intervention to improve mental health literacy amongst young people aged 12–14 in the UK
Source: Front Psychiatry. 2025 Jul 21;16:1546109. doi: 10.3389/fpsyt.2025.1546109 (PMC12319005; doi:10.3389/fpsyt.2025.1546109)
Supplement: Supplementary file 1 [file SupplementaryFile1.docx]

Supplementary Material

# Supplementary Tables

**Supplementary Table 1.** Suggested adaptations to existing app content.

| **Suggested changes** | **Priority for current project** | **Implemented?** |
| --- | --- | --- |
| Advisory group to approve the final script to ensure language is appropriate and reflects young people’s experiences. | Yes | Yes |
| Include greater gender diversity by removing the two gender options so the character is gender neutral. | Yes | Yes |
| Have greater customisation in the central character (full range of skin colours, ages, hair styles/colours, height, weight, piercings, outfits, disabilities e.g. option to include wheel chair/air tubes etc). | Yes | Yes |
| Allow customisation in terms of objects/hand held accessories for the main character (e.g. camera, phone and other relevant objects that could be included with the character) | Yes | Yes |
| Allow character to identify with different sexualities by having pride flags as options. Could link above to handheld accessories above (e.g. characters hold a pride flag). | No | No |
| Increase diversity and customisation in non-playable characters (e.g. age, gender, physical appearance, non-binary options) | Yes | To an extent. |
| Increase customisation in backgrounds (e.g. not all young people have a bedroom to themselves, make messier (clothes on the floor), customise colour scheme). | No | No |
| Have spoken option and allow customisation of background colours to improve access from neuro-diverse groups. | Yes | Not possible within current funding |
| Posters in room with positive mental health messages. It’s ok not to be ok. | No | No |
| Make street background look more like UK streets (include urban and rural options). Include different plants, more trees. | Yes | Yes |
| Professional rooms are more clinical in the UK | Yes | Yes |
| Make classrooms/professional rooms reflect the UK experience (e.g. no curtains, no separation of desks, just long rows. Chairs are ok but there should be no pull out under desk. Desks should all be looking forward. Include an interactive white board, no blackboard or TV. Consider having other school settings (dining room, social areas, student support rooms). A hub room (counselling room in schools) could be added. | Yes | Yes |
| Add toys to younger sibling’s room. | No | No |
| Teacher’s outfit not applicable to the UK. | Yes | Yes |
| Add more light to the evening rooms. | Yes | Yes |
| De-americanise some of the backgrounds and characters (psychiatrist/professional backgrounds) | Yes | Yes |
| Link mini games (google/networking) to existing UK resources (Kooth, 42^nd^ street, young minds, mind charity including those that allow peer interaction etc.). Update webpages in the web browser game, they look dated. Will this include skills to assess the validity of websites and how to assess their credibility? | Yes | Yes |
| Include activities to take off line at the end of each chapter to undertake with family/friends. Remind players to take strategies out of the game. Input a to do list that players can take off line. | Yes | Yes |
| For the mini games, could there be a brief overview of why strategies help with mental health? Could they be saved somewhere so the player can access after the chapter has been finished? | Yes | To an extent |
| Could the journal be customisable and one be generated for the player too? | No | No |
| Include reflexive exercises at the end of each chapter to consolidate learning. | Yes | To an extent |
| Allow young people to download a picture of the central character to share on social media or a video of gameplay. | No | No |
| Include notifications to prompt use of the app. | No | No |
| Have badges/stickers/stars to demonstrate progress and reward interaction with app. And/or certificate to download after completion. | Yes | Not possible within current funding |
| Quizzes with leader boards. | No | No |
| Have a diary option with emojis to track emotions over time. | No | No |
| Network game – include more options and have non-human options (e.g. hobbies/valued activities). See above for range of different family members, friends and professionals. | Yes | Yes |
| Distraction game to include hobbies/valued activities. | Yes | Yes |
| Provide a brief overview of each chapter before it launches so young people can be clear about what topics it covers. | No | To some extent |
| Google game should link to existing UK resources to promote access to valid information. | Yes | Yes |
| Need safeguarding building in. This could include a button about needing help or feeling distressed which brings up people to speak to or resources to access. | Yes | Yes |

**Supplementary Table 2.** Suggestions for additional chapter.

| **Suggested things to include** | **Agreed a priority for this study?** | **Has this been implemented?** |
| --- | --- | --- |
| Should not focus on diagnostic categories. | Yes | Yes |
| Separation of mental illness and normalising the emotional ups and downs associated with everyday life. | Yes | Yes |
| Come out of home setting. Be based in the community. | Yes | Yes – school and local community settings. |
| Underpin with Dialectical behaviour therapy or completing the stress cycle. | No | No |
| Multi-player options. | Yes | Not possible in the current funding. |
| Lighter focus – managing day to day stresses/anger and promoting wellbeing. | Yes | Yes |
| Need to normalise and help understand every day experiences. The role of social relationships was prioritised especially with peers. | Yes | Yes |
| Normalise a range of emotions, challenge ideas of how you ‘should’ be feeling. | Yes | Yes |
| Build resilience/self-confidence by highlighting the importance of sleep, social connections, valued activities (exercise, social interaction etc.) and understanding negative influences. | Yes | Yes |
| Cover self-care and healthy living. | Yes | Yes |
| Include park/nature/woodland/cafes/community venues in the new chapter. | Yes | To some extent |
| Value difference and uniqueness. | Yes | Yes |
| What are healthy/unhealthy relationships? What is a friendship? | Yes | To some extent |
| Focus on what YP has or what they are grateful for – interactive element to put notes in a jar of things that they are grateful for which they can revisit and/or share. | Yes | Yes |
| Understanding what is happening in the body when we feel emotions. And mentioning hormone changes during puberty to normalize feelings that are new for them | Yes | Yes |
| Helping young people to recognise unhelpful thinking habits and triggers of stress. | Yes | Yes |
| Other topics to consider (loneliness/racism/refugees/body image) | No | For future work |
| Include materials such as videos/animations detailing game play to promote engagement. | No | No |
| Explore character who seems well on the surface (performing well at school etc) but is struggling underneath and maybe masking struggles. | Yes | Yes |
| Labelling emotions mini-game. | Yes | Yes |
| Mindfulness/deep breathing exercises. | Yes | Yes |
| How to avoid catastrophising | No | No |
| Video journaling. | No | No |
| Exercises around when help is needed and what can be self-managed. And where to go for what can not be self-managed | Yes | To some extent |
| Game - What to say to someone if they are experiencing difficulties. | No | No |

# Supplementary Figures

**
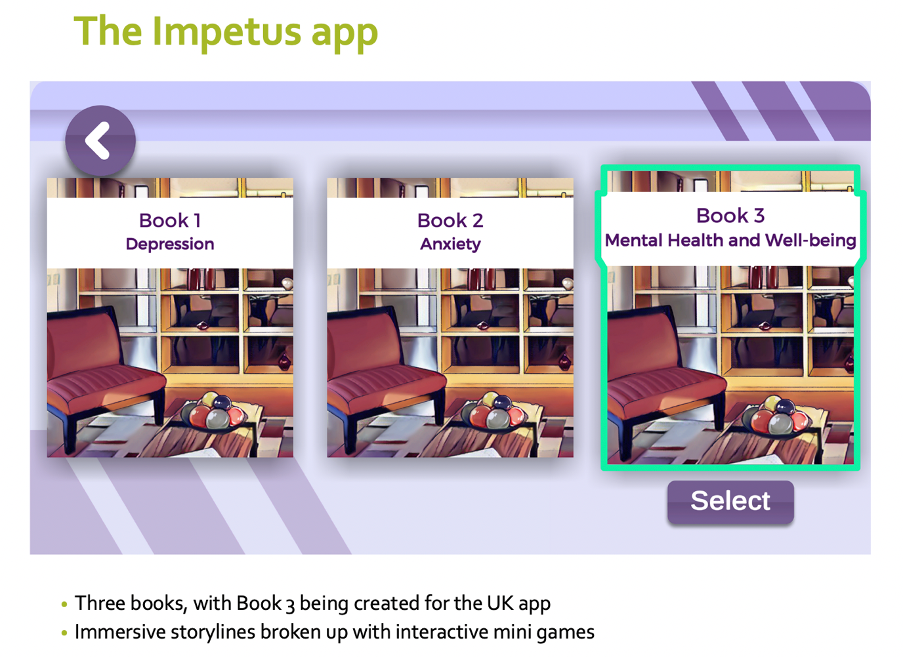
Supplementary Figure 1.** IMPeTUs chapter/book selection including two existing chapters and a co-created third chapter.


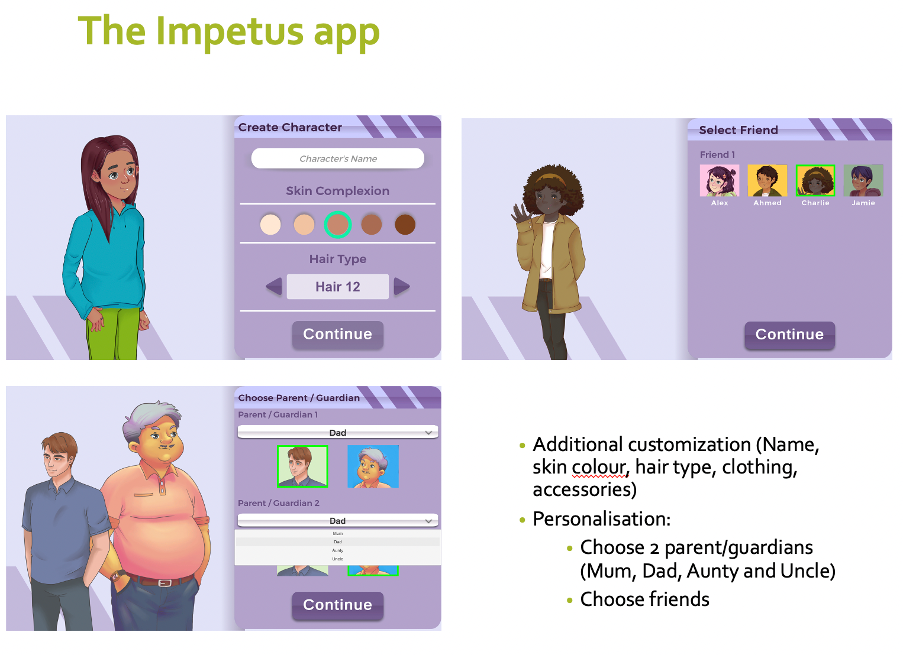


**Supplementary Figure 2.** Co-adapted IMPeTUs character, family, and friend customization.

**
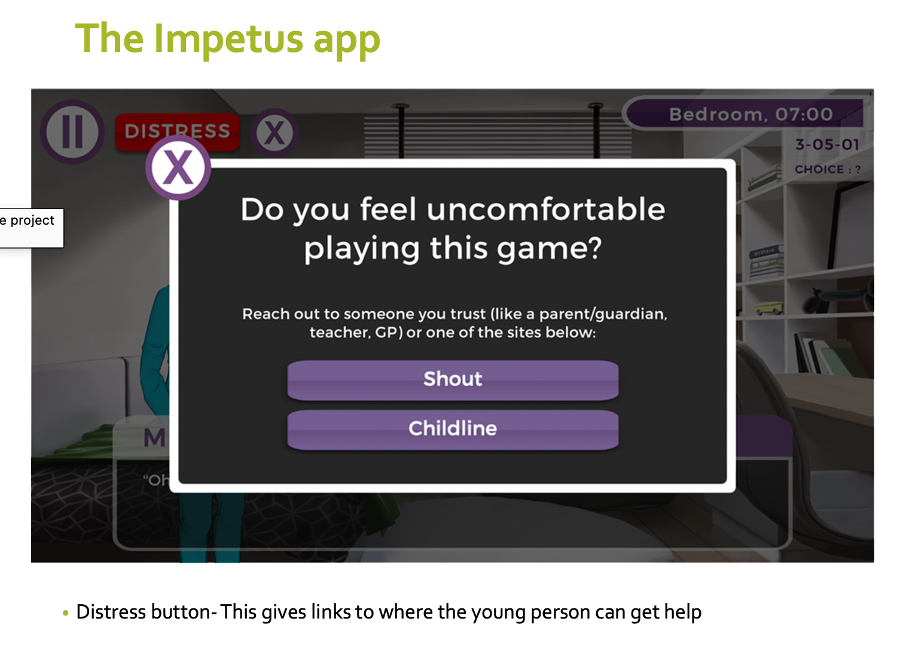
**

**Supplementary Figure 3.** Co-adapted IMPeTUs in-application pop-up when distress button is pressed.

**
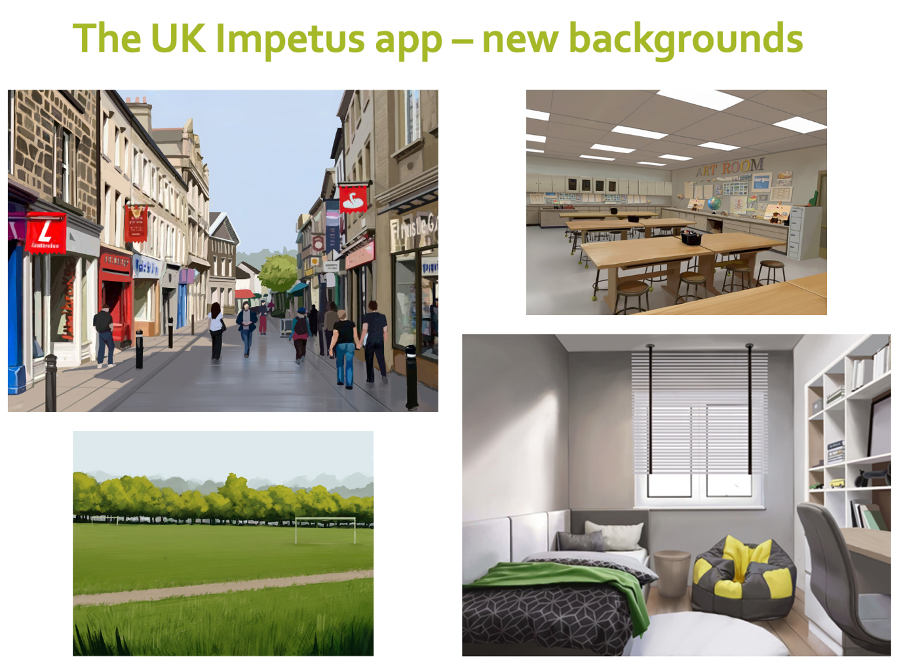
**

**Supplementary Figure 4.** New backgrounds included in UK version of IMPeTUs app.
